# Silicoaluminates as “Support Activator” Systems in Olefin Polymerization Processes

**DOI:** 10.3390/ma3021015

**Published:** 2010-02-03

**Authors:** Vanessa Tabernero, Claudimar Camejo, Pilar Terreros, María Dolores Alba, Tomás Cuenca

**Affiliations:** 1 Departamento de Química Inorgánica, Universidad de Alcalá, Campus Universitario, E-28871 Alcalá de Henares, Spain; E-Mails: vanessa.tabernero@uah.es (V.T.); cclaudimar@hotmail.com (C.C.); 2 Instituto de Catálisis y Petroleoquímica, CSIC, Cantoblanco, 28049 Madrid, Spain; E-Mail: pterreros@icp.csic.es (P.T.); 3 Instituto de Ciencia de Materiales de Sevilla, Universidad de Sevilla, CSIC, 41092, Sevilla, Spain; E-Mail: alba@icmse.csic.es (M.D.A.)

**Keywords:** montmorillonite, SBA-15, ethylene, zirconium, acidity, polymerization

## Abstract

In this work we report the polymerization behaviour of natural clays (montmorillonites, MMT) as activating supports. These materials have been modified by treatment with different aluminium compounds in order to obtain enriched aluminium clays and to modify the global Brönsted/Lewis acidity. As a consequence, the intrinsic structural properties of the starting materials have been changed. These changes were studied and these new materials used for ethylene polymerization using a zirconocene complex as catalyst. All the systems were shown to be active in ethylene polymerization. The catalyst activity and the dependence on acid strength and textural properties have been also studied. The behaviour of an artificial silica (SBA 15) modified with an aluminium compound to obtain a silicoaluminate has been studied, but no ethylene polymerization activity has been found yet.

## 1. Introduction 

Currently, polymer and plastics based on polyolefin materials play an important role in the materials industry and in society. Usually, the chemical systems used for α-olefin polymerization catalysis in the synthesis of the polyolefin materials combine a metal complex as a precatalyst system with a Lewis acid species as cocatalyst. The most studied precatalyst systems for this purpose are generally formed by group IV metal compounds, which are transformed into the catalytically active species in the presence of a cocatalyst [1,2,3]. Methylaluminoxane (MAO) is obtained by partial hydrolysis of trimethylaluminium (TMA) [4,5,6] and constitutes the industrial base of cocatalyst products for activation of metallocene precatalysts in olefin polymerization. Due to its Lewis acidity, MAO can produce ligand exchange from the precatalysts and subsequent ligand abstraction from the metallocene complex to form a cationic complex as the active species for the polymerization reaction. However, the exact role of MAO has not yet been fully elucidated, due in part to its complex structure [7,8,9,10]. Different cocatalysts have been developed to control the activity, selectivity, molecular weight and other olefin polymerization catalytic features [10]. In recent years, clay minerals [11] or zeolite-supports [7,8,9,12,13,14,15] have received attention as alternative cocatalysts [16,17] in olefin polymerization reactions.

Another approach has focused on natural 2:1 phyllosilicates which have been used as adsorbents, ion exchangers, solid catalysts or supports in heterogeneous processes [18,19,20,21]. Recent publications have shown that modifications to the structure or the acidic properties of the supports make them good cocatalysts in different chemical processes [16,22,23,24]. 

Herein we propose the use of an acid-treated commercial montmorillonite (MMT), which, after modification with conventional trialkylaluminium derivatives AlR_3_ [R = Me (TMA), Et (TEA)], provides a new material which can act simultaneously as cocatalyst (like MAO) and as carrier when combined with a metallocene type complex ZrCp*Cp’Cl_2_ [where Cp* = C_5_Me_5_; Cp’ = C_5_H_4_(SiMe_2_CH_2_CH=CH_2_)] to give species active in olefin polymerization. Clay minerals used as cocatalyst and simultaneously as catalyst support have been considered as support activators [23].

Many advantages in combining the two fields could be highlighted, for example, the development of systems without an expensive organoaluminoxane compound (*i.e*., MAO free) but with high catalytic activity and low cost would be a desirable objective. To get a better knowledge of the nature of the active species and the possible activation mechanism, we have studied the acidity and textural properties of these materials in order to correlate them with their activity in the polymerization reaction. 

Alternatively, a mesostructured silica has been synthesized in the laboratory (SBA 15) [25] and modified with aluminium to give an “aluminosilicate” material. The behaviour of this mesostructured material used as support activator for ethylene polymerization with a zirconium complex acting as precatalyst has been also studied.

## 2. Results and Discussion 

### 2.1. Characterization of Support Activators

The properties of two commercial acid-treated clays of the K series (K10, K30) have been studied by μXRF analysis and are listed in Table 1. The results are in accordance with analogous studies described in the literature [11,26] and show that acid treatment of the clay replaces the initial interlayer cations with protons (the pH of K10 and K30 is between 3–4) and causes disaggregation of the sheets, giving rise to a delaminated structure accompanied by silica formation [27,28,29].

**Table 1:** Chemical analysis (%) of commercial acid treated MMT clays.

	K10	K30
**SiO_2_**	82.39	80.34
**Al_2_O_3_**	13.84	15.44
**MgO**	1.41	1.41
**Fe_2_O_3_**	0.42	0.63
**TiO_2_**	0.13	0.16
**Na_2_O**	0.94	0.90
**K_2_O**	0.71	1.00
**CaO**	0.16	0.12

**Figure 1:**
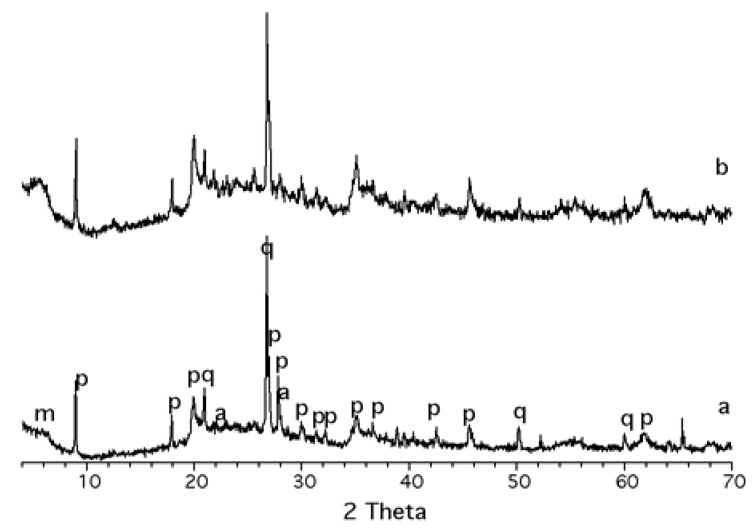
XRD of acid treated montmorillonites: (a) K10; (b) K30. m = montmorillonite (PDF 3-0015); p = phengite 2M_1_ (PDF 76-0928); a = albite (PDF=41-1480); q = quartz (PDF 78-2315).

The XRD patterns of K10 and K30 are shown in Figure 1. For both samples, the reflection of the diffractograms match with the diffraction pattern of a mica, phengite-2M_1_ (PDF 76-0928), with a basal space of 9.92 Å, which is typical of collapsed 2:1 phyllosilicates. At 5.82° 2θ, a wide 001 reflection corresponding to remaining montmorillonite (PDF 3-0015, marked with *m* in Figure 1) is observed, reflecting a quite disordered stacking in the layers with a basal space of 15.17 Å. 

Additionally, some reflections that match with the diffraction patterns of albite (PDF 41-1480, marked with *a* in Figure 1) and quartz (PDF 78-2315, marked with *q* in Figure 1) are observed, this structural behaviour is more evident in K10 than in K30. Finally, the XRD patterns exhibit a prominent background between 20° and 30° 2θ, corresponding to amorphous phases [26]. 

**Figure 2:**
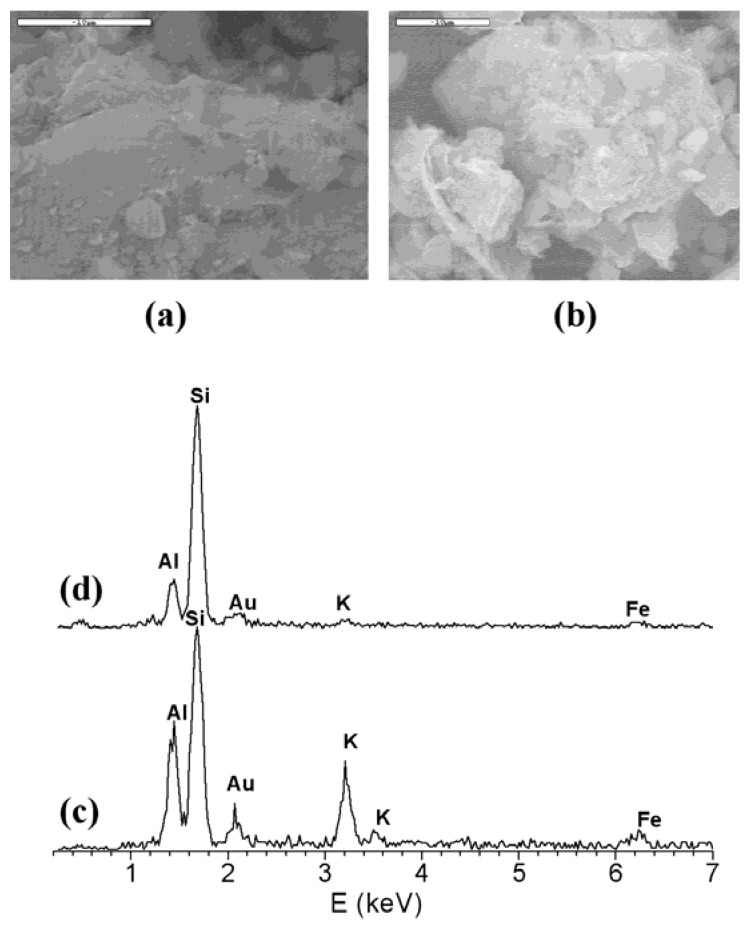
SEM microphotographs and EDX spectra of acid treated montmorillonites lamellar particles (a), (c): K10; (b), (d): K30.

The analysis of the samples by scanning electron microscopy reveals that the majority of the particles have a lamellar morphology (Figure 2) with a composition compatible with 2:1 phyllosilicate with K^+^ as interlayer cation, and they must correspond with the mica phase observed by XRD. Additionally, some small block particles with a heterogeneous chemical composition, but with a Si content higher than those shown by the lamellar particles, are observed.

**Figure 3:**
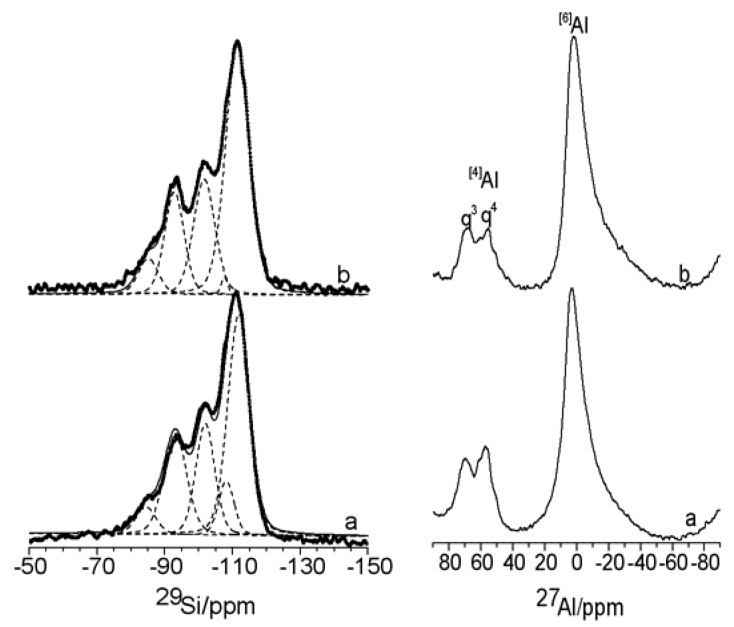
^29^Si- (left) and ^27^Al- (right) MAS-NMR spectra of acid treated montmorillonites (a): K10; (b): K30.

Short-range analysis of the initial montmorillonites using ^29^Si- and ^27^Al-MAS-NMR (Figure 3) has been performed. The ^29^Si spectra (Figure 3, left) are characterized by five deconvoluted signals ranging between -60 and -150 ppm which correspond to Si environment Q^3^ and Q^4^, following the Liebau classification [30]. The signal at *ca.* -85 ppm corresponds to the Q^3^(3Al) environment of the mica [31,32] and contributes 5.2% and 6.5%, for K10 and K30 respectively, to the total spectrum. Notably, the signal shifts from -84.4 ppm for K10 to -85.1 ppm for K30, possibly due to different layer charges in each mica structure [33]; as the layer charge of smectite increases the signal shifts to higher frequency. The signal at *ca.* -108 ppm corresponds to quartz [34] and the contribution is greater in K10 (7.8%) than in K30 (0.9%) as previously reported by XRD. Finally, the three signals at *ca.* -111 ppm, -102 ppm and -93 ppm correspond to Q^4^(4OSi), Q^4^(3OSi,OH) and Q^4^(2OSi,2OH) environments [34]. The signal at *ca.* -93 ppm is consistent with overlapping Q^4^(2OSi,2OH) and Q^3^(0Si) environments of the montmorillonite [33,35]. 

The ^27^Al-MAS-NMR spectra (Figure 3, right) show three signals at *ca.* 0 ppm, due to octahedral coordination, with the other two signals in the range between 70 an 50 ppm, due to tetrahedral coordination [36,37,38]. The signal at *ca.* 0 ppm and at *ca.* 70 ppm (q^3^) is typical of aluminium in dioctahedral 2:1 phyllosilicates (montmorillonite and mica phases) and the signal at 55 ppm (q^4^) results from the tetrahedral aluminium in feldspars [34]. The intensity of the q^4^ signal *vs.* the q^3^ signal is higher in montmorillonite K10.

These commercial MMT have been later modified by treatment with aluminium compounds (AlR_3_:TMA and TEA) to obtain the support activator material and to understand the role of the R alkyl group. The AlR_3_ compound has different functions; as a consequence of aluminium incorporation, the Brönsted and Lewis acidity of the original clay might be modified; in addition, it prevents the catalytic sites from being poisoned by adventitious water or by hydroxyl groups present in the clay. Finally, when using a non-alkylated zirconocene compound such as ZrCp*Cp’Cl_2_ even if the acidity of the support enables the formation of cationic species, in order to obtain the active species, the starting complex must be alkylated.

**Figure 4:**
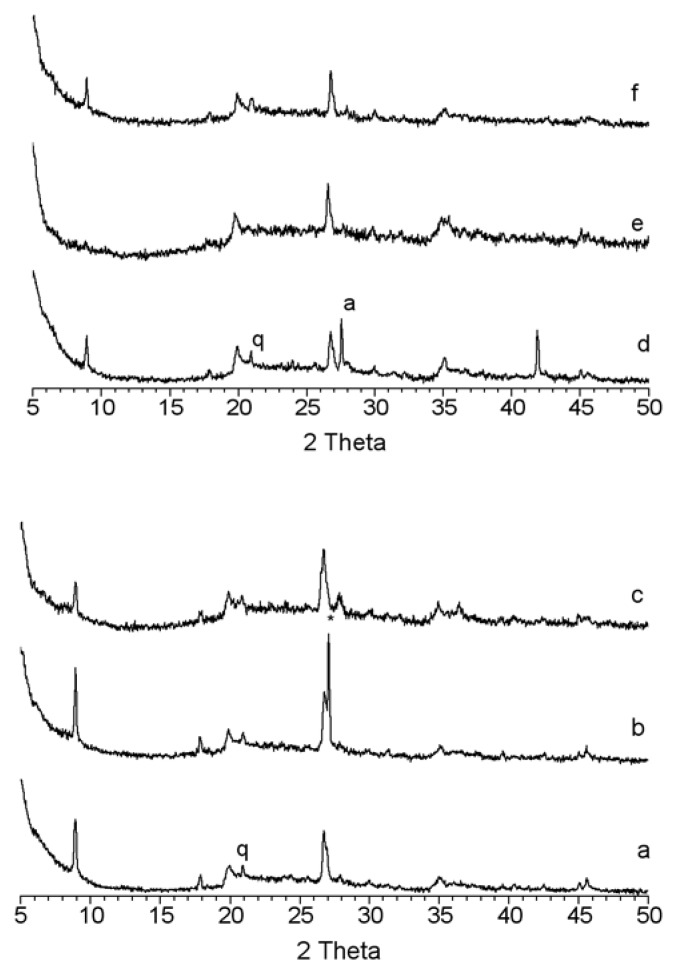
XRD in inert atmosphere of acid treated montmorillonites (a) K10; (b) K10/TMA; (c): K10/TEA; (d) K30; (e) K30/TMA; (f) K30/TEA; a = albite; q = quartz.

**Figure 5:**
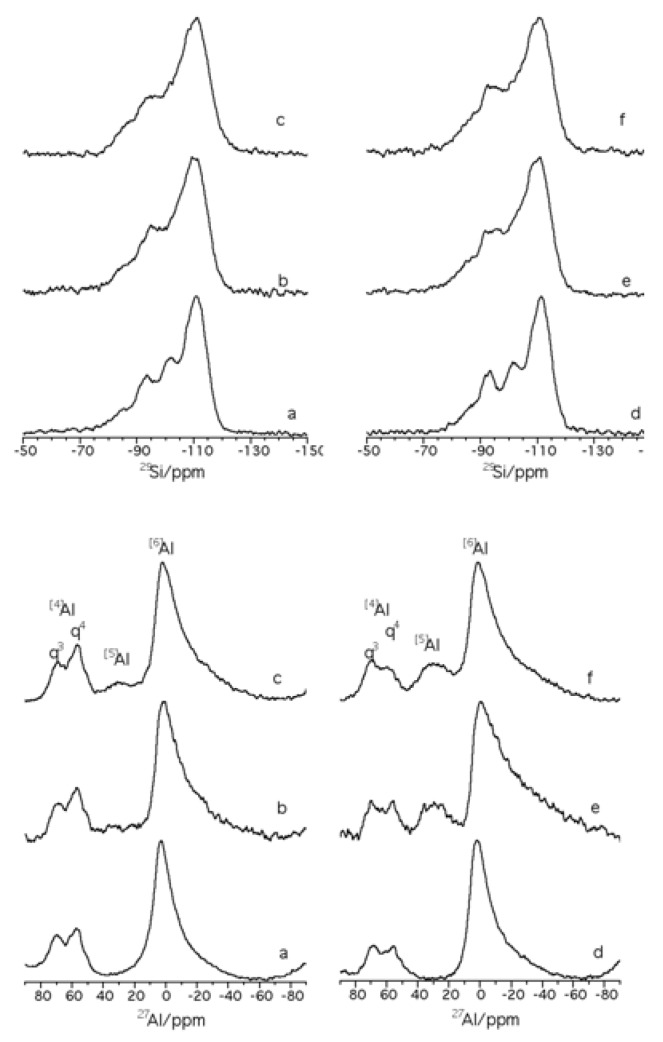
^29^Si- (top) and ^27^Al- (bottom) MAS-NMR spectra of acid treated montmorillonites (a) K10; (b) K10/TMA; (c) K10/TEA; (d) K30; (e) K30/ TMA; (f) K30/TEA.

The aluminium compounds were attached to MMT by alkane elimination through reaction with the hydroxyl groups of the clay or with the protons of the cations in the interlayers; gas evolution is observed during the process. Because an excess amount of AlR_3_ compound was used in the preparation, the solid obtained was washed three times (see Experimental section) and vacuum dried. The resulting solid was stored at low temperature in a dry box. Evidence of reaction is shown in the FT-IR spectra which show in all cases a significant decrease in the intensity of the bands at 3,440 cm^-1^ and 1,639 cm^-1^ for the water of hydration and the appearance of a signal at 2,900 cm^-1^ assigned to the C-H vibrations due to the alkyl groups of the attached aluminium compound [16,39]. µXRF analysis (Table 2) also demonstrates that aluminium was efficiently incorporated into the silicate material during this process, and this observation was further confirmed by MAS-NMR analysis (Figure 5). The treatment of montmorillonites with the aluminium compound, AlR_3_ do not provoke great changes at long-range as demonstrated by XRD at dry ambient temperature (Figure 4). In general, changes in the basal space of mica phase have not been detected, indicating that the aluminium complex is not placed in the interlayer space. However, the treatment causes a disorder in the layer as demonstrated by the intensity decrease of the 9.5° 2θ reflection of the montmorillonites treated with AlEt_3_ (Figure 4c and Figure 4f); this decrease is absent in K30/TMA (Figure 4e). After the treatment, the reflections of impurities (quartz and albite) decrease and a new reflection is observed in K10/TMA corresponding to Al_2_O_3_ (PDF 26-0031). 

The short-range order analysis of ^29^Si and ^27^Al nuclei (Figure 5) reveals that there is no significant difference in the ^29^Si-NMR profiles (Figure 5, top), which are characterized by the same set of signals centred at the same frequencies but with a different relative intensity. The montmorillonites treated with the AlR_3_ complex cause a decrease in the Si of mica (more significant after treatment with the TEA complex than with the TMA complex) and an increase of the Si of quartz (more significant in the treatment with TMA complex than with the TEA complex). The ^27^Al-NMR spectra (Figure 5, bottom) reveal the presence of a new signal at ca. 30 ppm due to pentacoordinate aluminium [34] which could result from the interaction of the AlR_3_ with the hydroxyl group of silicate surface thorough Lewis acid sites. The pentacoordinate aluminium signal is more prominent in K30 than in K10.

The amount of AlR_3_ adsorbed onto the silicate surface has been evaluated thorough µXRF and the results are summarized in Table 2. The amount of aluminium absorbed is higher in K30 than in K10 and, in general, the TEA complex is more easily adsorbed than TMA complex. These results support the presence of the new aluminium signal in the ^27^Al-NMR spectra.

**Table 2:** Aluminium attached on the commercial MMT obtained by µXRF.

	^†^g(Al_2_O_3_)_compl._/100g K10	^††^g(Al_2_O_3_)_compl._/100g K30
Initial	--	--
TMA	17.63	25.85
TEA	18.02	29.95

### 2.2. Ethylene Polymerization

These support activators combined with the zirconium compound ZrCp*Cp’Cl_2_ gave catalytic activity for ethylene polymerization (Table 3). Potential complicated effects (e.g., catalyst autosupportation) are not detected by using the pending allyl group bounded to the cyclopentadienyl ring. Studies with other metallocene derivatives are in progress. It is important to point out that before the polymerization experiment, the mixture of support activator and zirconium complex was washed twice to ensure that the possible complexes in the homogeneous phase of the supernatant have been eliminated and that they are not the species responsible for the catalytic activity. This activity reveals that organic aluminium compounds anchored on MMT generate some species effective as activators of the zirconocene compound for ethylene polymerization. The system based on K10/TMA/ZrCp*Cp’Cl_2_ shows the higher activity, whereas the system based on K30/TMA/ZrCp*Cp’Cl_2_ is the less active. In view of these preliminary results, we suggest that the amount of aluminium retained in the matrix (evaluated by μXRF analysis) seems not to be the only parameter determining activity, but the environment and the acidity (see discussion below) of these atoms must also influence their behaviour when they act as cocatalysts.

DSC measurements were conducted for the polymers obtained (Table 3). A single peak (about 134 °C) was observed for all samples in the DSC profiles suggesting HDPE. 

**Table 3:** Ethylene polymerization using modified MMT as support activators.

Entries	Support-Activator	Activity	Tm(°C)
1	K10/TMA	19,143	133.7
2	K10/TEA	7,143	133.4
3	K30/TMA	3,857	134.0
4	K30/TEA	15,000	134.5

^a^ Polymerization conditions: catalyst ZrCp*Cp’Cl_2_: 7.10^-5^ moles, 2.5 g of support-activator, temperature = 50 °C; 1 mL TIBA, 50 mL of toluene; 1 atm. of ethylene pressure; time = 60 min.; ^b^ Activity for ethylene polymerization, gPE/mmol Zr·h·atm; ^c^ Determined by DSC.

Under the same conditions, polymerization of ethylene was carried out using a post-synthesis alumination SBA15 [40] as support activator. The procedure of alumination was carried out using TMA, although when the resulting solid was combined with ZrCp*Cp’Cl_2_, the system was inactive in ethylene polymerization.

### 2.3. Studies on the Nature of Catalytically Active Species

In order to gain a better knowledge of the nature of the active species in these polymerization reactions, we examined the behaviour of montmorillonites as support activators for olefin polymerization in the presence of ZrCp*Cp’Cl_2_. However, the activation mechanism in these systems remains unclear. One interesting question therefore arises: what type of interaction can occur between the precatalyst and the support activator in order to generate the active centre?

**Scheme 1:**
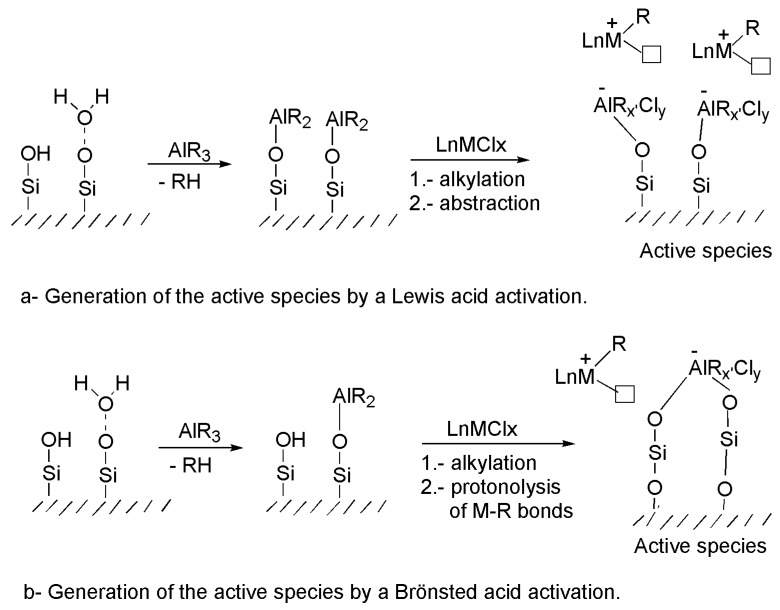
Different activation pathways of precatalyst complexes considering the different nature of the acidic sites in the support activator.

Recent investigations on supported metallocene catalysts based on smectite supports have been reported [23,24]. It is known that the acidic properties of these materials may influence the type of interaction with the metallocene complex and its activation process, with consequent effects on overall productivity [41,42,43]. These clay materials have Lewis and Brönsted acidic sites and their actuation to generate the active species could be argued in two ways, similar to the homogeneous systems. It is well known that the Lewis acidity of MAO arising from aluminium centres can produce ligand exchange and subsequent alkyl/halide abstraction in activating metal complexes (organo-Lewis-acidic cocatalyst). This type of activation process could be applied to clay material as cocatalysts. In this case, the clay materials should be considered, initially, as a modified MAO (or analogous alkylaluminoxane) [13,14,17,44,45], which are formed by the reaction of the alkylaluminium compound with residual water molecules or with the hydroxyl surface groups on MMT (Scheme 1a). 

For homogeneous catalysis, an alternative activation process involves protonolysis of the M-R bonds, in non-coordinating solvents, of the precatalyst using a Brönsted-acidic cocatalyst (e.g. trialkylammonium salts) to give finally a cationic derivative as active species [10]. In this sense, the catalytic behaviour of the clay should be understood due to the presence of protons located between the MMT silicate layers to balance the total charge deficiency of the clay or due to the hydroxyl groups in the surfaces of the clay. In this case, the metallocene can be immobilized on the material by a reaction consisting of protonolysis of the precatalyst M-R bonds to generate the highly electrophilic cationic species for polymerization (Scheme 1b).

**Figure 6:**
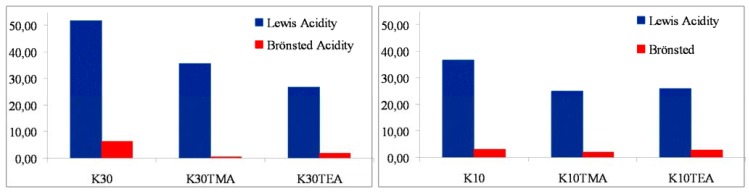
Evaluation of acidity from the IR/FT spectra of samples after pyridine sorption and degassed at 200 °C.

The common feature in these models is the generation of a cationic or cationic-like active species for the polymerization reaction. The Lewis and Brönsted acidities, for our catalytic systems based on commercial acid-treated clays of the K series (K10, K30), have been determined using pyridine as a probe molecule by monitoring the bands in the range of 1,350–1,600 cm^-1^. The characteristic adsorption band for pyridine absorbed on Brönsted acid sites (Bpy) appeared at 1,540 cm^-1^ and that due to Lewis acidity (Lpy) at 1,450 cm^-1^ [19,46]. The ratio between these two acidic sites is indicated by Bpy/Lpy (Figure 6). The experimental data permit us to clearly conclude that the main fraction of the acid sites, detected in our modified aluminium MMT system, is due to Lewis acidity. The FTIR spectra show that K30 has a larger peak area at 1,492 cm^−1^ than K10, indicating that K30 has more acid sites (or active sites) than K10. These results point to Lewis acidity being responsible for the activity of these MMT/Al species when they used as support activators for ethylene polymerization [47].

Effectively, the studies of acidity on similar mesostructured materials, based on SBA15 [40] aluminated species, show fewer accessible Lewis sites [40,48,49,50]. This finding is consistent with the inactivity exhibited by these systems as support activators in our experimental ethylene polymerization studies. Textural properties might also affect catalytic activity. In this way, differences in the pore size and volume may cause problems with precatalyst anchoring or monomer diffusion, decreasing or even cancelling the catalytic activity of the SBA15 samples [51,52,53].

## 3. Experimental Section 

### 3.1. Reactants 

All chemicals were manipulated under an inert atmosphere using Schlenk techniques. Solvents were predried by standing over activated 4 Å molecular sieves and then purified by distillation under argon, employing the appropriate drying/deoxygenated agent, before use. K10, K30, solution of AlMe_3_ in toluene, and solution of AlEt_3_ in hexane, were purchased from Aldrich or Fluka. Zr(C_5_Me_5_)[(C_5_H_4_)SiMe_2_(CH_2_CH=CH_2_)]Cl_2_ [54] was prepared according to literature procedures. Ethylene was polymerization grade and passed through a purifying cartridge (Alltech 81015) before use.

### 3.2. Characterization

#### 3.2.1. Characterization of Zirconium Complex

NMR spectra were recorded at the University of Alcalá on a Bruker AV400 instrument. Resonances were measured relative to solvent peaks considering ^1^H and ^13^C TMS δ = 0 ppm at 400.13 (^1^H) and 100.60 (^13^C) MHz. Elemental analyses were obtained on a Perkin-Elmer Series II 2400 CHNS/O analyzer. 

#### 3.2.2. Characterization of supports and support activators

X-ray diffraction (XRD) patterns were obtained at the CITIUS X-ray laboratory (University of Sevilla) on a Bruker D8 Advance instrument equipped with a Cu Kα radiation source operating at 40 kV and 40 mA. Diffractograms were obtained in the 2θ-range 3–70° with a step size of 0.05° and a time step of 3.0 s. The XRD diffraction pattern of the Montmorillonite complex was obtained on a Bruker D8 Advance instrument, at the CITIUS X-ray laboratory (University of Sevilla), fitted with an ambient camera (Anton Paar XRK 900, Austria) and a position-sensitive detector (Bruker Vantec PSD, Germany). Diffractograms were obtained in the 2θ-range 3–70° with a step size of 0.05° and a time step of 3.0 s. The micro X-ray fluorescence (µXRF) analysis was carried out in the Centro de Investigación, Tecnologías e Innovación of University of Sevilla (CITIUS). µXRF measurements were performed in an EAGLE III (EDAX) energy dispersive micro X-ray fluorescence spectrometer equipped with an Rh X-ray tube, 300 micron monocapillary optics, a CCD camera and an 80 mm^2^ Si (Li) detector. Surface scans of 0.5 cm^2^ were performed under vacuum with measurement times of 150 seconds. Use of the fundamental parameter quantification made automated analyses routine. Single-pulse (SP) MAS-NMR experiments were recorded by the Spectroscopy Service of ICMS (CSIC-US, Sevilla) using a Bruker DRX400 spectrometer equipped with a multinuclear probe. Powdered samples were packed in 4-mm zirconia rotors and spun at 10 kHz. ^1^H-MAS spectra were obtained using a typical π/2 pulse width of 4.1 μs and a pulse space of 5 s. ^29^Si-MAS-NMR spectra were acquired at a frequency of 79.49 MHz, using a pulse width of 2.7 μs (π/2 pulse length = 7.1 μs) and a delay time of 3 s. ^27^Al-MAS-NMR spectra were recorded at 104.26 MHz, using a pulse of π/20 of 1.1 μs and a delay time of 0.5 s. The chemical shift values are reported in ppm with respect to tetramethylsilane for ^1^H and ^29^Si and to 0.1 M AlCl_3_ solution for ^27^Al. The morphology and chemical composition of the samples were analyzed by Scanning electron microscopy (SEM), at the Microscopy Service of the Instituto Ciencia de los Materiales de Sevilla (CSIC-US), with a Scanning Electron Microscope (JEOL JSM 5400) equipped with a LINK Pentafet probe and ATW windows for Energy Dispersive X-ray Analysis (EDX). The Lewis and Brönsted acidity have been determined using pyridine as a probe molecule by monitoring the bands in the range of 1,350–1,600 cm^-1^. FTIR spectra in the range 1,400–1,700 cm^−1^ were recorded by the Spectroscopy Service of ICMS (CSIC-US, Sevilla) using a Nicolet spectrometer (model 510P) with a nominal resolution of 4 cm^-1^. 

#### 3.2.3. Characterization of polymers

The thermal properties of the samples were studied in a Perkin Elmer DSC 6 (University of Alcalá) instrument calibrated by measuring the melting point of indium. 5–10 mg each of the dried polymer were fused into standard aluminium pans and measured using the following temperature programme for polyethylene samples: first a heating phase (10 °C/min) from 50 to 200 °C, followed by a cooling phase (–10 °C/min) to 50 °C. The peak maximum of the second heating curve was indicated as the melting point (*Tm*). 

### 3.3. Preparation of the Support Activator

AlMe_3_ solution was added to a suspension of the desired MMT (relation 6.6 × 10^-3^ mol of aluminium compound/g of MMT) in aprox. 50 mL of toluene. Heat was generated and accompanied by the generation of a gas. After completing the dropwise addition, the reaction mixture was maintained at room temperature for three hours with vigorous stirring, then it was filtered and the resulting solid was washed twice with toluene (40 mL each time) and a third time with 40 mL of hexane. Finally the solid was dried and stored in the dry-box at low temperature.

The same procedure was followed with AlEt_3_ but using hexane as solvent (50 mL) for the reaction rather than toluene. In this case the two first washes were made with hexane (40 mL each) and the third wash that was made with 40 mL of toluene.

### 3.4. Polymerization procedure

In a dry box, the desired amount of the support activator (2.5 g) was suspended in toluene and the catalyst (7 × 10^-5^ moles) was added via syringe. Then the mixture was allowed to react for one hour at room temperature. After this time the supernatant was filtered and the resulting solid was washed for 30 min with toluene (20 mL). Again the supernatant was filtrated off and the dried solid was placed in the polymerization reactor charged with a magnetic stirrer, 50 mL of toluene and 1 mL of TIBA. This catalyst slurry was taken out of the dry box and put in an oil bath. When the desired temperature was reached, ethylene was introduced at 1 atm. and gas supply was constant during the process. At the end of the reaction time, the remaining ethylene was released and polymerization was stopped with EtOH/HCl. The polyethylene (PE) was recovered by filtration. The catalytic activity was calculated from the yield of PE and the amount of metallocene complex used.

## 4. Conclusions 

Commercial MMT (K10 and K30) modified with alkyl aluminium compounds (AlMe_3_ or AlEt_3_) generate effective support activators when combined with a zirconium complex for ethylene polymerization. A systematic study of the structural parameters of the support activators and a correlation study of their activity have been carried out. The system K10/TMA/ZrCp*Cp’Cl_2_ showed the most catalytic activity. The important role of acidity in the support activator shows that activity for olefin polymerization is a complex balance of different factors, and the type of interaction of the AlR_3_ with the surface plays a more important role than the global amount of the AlR_3_ absorbed. In summary, a successful application of a very inexpensive and “green” material based on MMT clays has been probed as a support activator system in olefin polymerization. We believe the most important achievement of this work is the development of a useful alternative α-olefin polymerization system to MAO.
